# Centrifugation Force and Time Alter CASA Parameters and Oxidative Status of Cryopreserved Stallion Sperm

**DOI:** 10.3390/biology9020022

**Published:** 2020-01-27

**Authors:** Giuseppina Marzano, Natalina Moscatelli, Mariangela Di Giacomo, Nicola Antonio Martino, Giovanni Michele Lacalandra, Maria Elena Dell’Aquila, Giuseppe Maruccio, Elisabetta Primiceri, Maria Serena Chiriacò, Vincenzo Zara, Alessandra Ferramosca

**Affiliations:** 1 Department of Mathematics and Physics E. de Giorgi, University of Salento, 73100 Via per Arnesano, Lecce, Italy; giuseppina.marzano@unisalento.it (G.M.); giuseppe.maruccio@unisalento.it (G.M.); 2 Institute of Nanotechnology, CNR NANOTEC, 73100 Via per Monteroni, Lecce, Italy; elisabetta.primiceri@nanotec.cnr.it (E.P.); mariaserena.chiriaco@nanotec.cnr.it (M.S.C.); 3 Scuola Superiore ISUFI (Istituto Superiore Universitario di Formazione Interdisciplinare), University of Salento, 73100 Via per Arnesano, Lecce, Italy; 4 Department of Biological and Environmental Sciences and Technologies, University of Salento, 73100 Via per Monteroni, Lecce, Italy; natalina.moscatelli@unisalento.it (N.M.); mariangela.digiacomo@unisalento.it (M.D.G.); vincenzo.zara@unisalento.it (V.Z.); 5 Center for Biomolecular Nanotechnologies @UNILE, Istituto Italiano di Tecnologia, Via Barsanti, I-73010 Arnesano, Lecce, Italy; 6 Department of Biosciences, Biotechnologies & Biopharmaceutics, University of Bari Aldo Moro, km3 Strada per Casamassima, 70100 Valenzano, Bari, Italy; mariaelena.dellaquila@uniba.it; 7 Department of Veterinary Sciences, University of Torino, Largo Paolo Braccini, 10095 Grugliasco, Torino, Italy; 8 Department of Veterinary Medicine, University of Bari Aldo Moro, km3 Strada per Casamassima, 70100 Valenzano, Bari, Italy; giovannimichele.lacalandra@uniba.it

**Keywords:** sperm motility, mitochondria, oxidative stress, DNA fragmentation, assisted reproductive technology

## Abstract

Conventional sperm selection techniques used in ARTs rely on centrifugation steps. To date, the different studies reported on the effects of centrifugation on stallion sperm motility provided contrasting results and do not include effects on mitochondrial functionality and different oxidative parameters. The effects of different centrifugation protocols (300× *g* for 5′, 300× *g* for 10′, 1500× *g* for 5′ and 1500× *g* for 10′ vs. no centrifugation) on motility and oxidative status in cryopreserved stallion sperm, were analyzed. After centrifugation, almost all motility parameters were significantly altered, as observed by computer-assisted sperm analysis. A polarographic assay of oxygen consumption showed a progressive decrease in mitochondria respiration from the gentlest to the strongest protocol. By laser scanning confocal microscopy, significant reduction of mitochondrial membrane potential, at any tested protocol, and time-dependent effects, at the same centrifugal force, were found. Increased DNA fragmentation index at any tested protocol and time-dependent effects at the same centrifugal force were found, whereas increased protein carbonylation was observed only at the strongest centrifugal force. These results provide more comprehensive understandings on centrifugation-induced effects on cryopreserved stallion sperm and suggest that, even at a weak force for a short time, centrifugation impairs different aspects of equine sperm metabolism and functionality.

## 1. Introduction

Assisted reproductive technologies (ARTs) consist of all procedures for handling female and male gametes and leading to embryo production aimed to overcome infertility in humans. Conventional sperm selection techniques used in human ARTs are density gradient centrifugation (DGC), which separates spermatozoa according to their density, and swim up (SU), which separates spermatozoa according to their motility [1,2]. Such procedures are also employed in the animal breeding industry to produce embryos to increase reproductive efficiency, to gain genetic improvement, and to preserve endangered animal species [3]. These techniques rely on centrifugation steps that are thought to cause damage to spermatozoa and are matter of concern in the ARTs field, especially in the case of poor quality sperm samples. Different sperm parameters have been investigated after preparation by these two conventional methods in several species. In human sperm, it was found that SU increased reactive oxygen species (ROS) levels in oligoasthenoteratozoospermic samples [4]. More specifically, centrifugation “per se” was responsible for human sperm decrease of motility over time, suggesting a sublethal damage [5], and morphological injury, such as a midpiece with disassembled and denuded axoneme ultra-structures [6]. Additionally, both DGC and SU were associated with chromatin fragmentation and ROS levels increase in normospermic samples [7], whereas only DGC led to a higher chromatin fragmentation in samples obtained from different patients undergoing ART cycles [8]. In the bull, DGC was shown to impair acrosomal membrane integrity [9]. In rats, centrifugation was reported to affect epididymal sperm motility, plasma membrane integrity and mitochondrial membrane potential (MMP) [10,11].

The horse is a suitable animal model for studying the efficacy of sperm selection methods and, due to the use of a wide range of ARTs [12,13,14], it is known as having translational relevance in human reproductive medicine. The horse is a seasonal breeder and the quality of horse semen is influenced by many factors, including season of year, the age, and frequency of ejaculation. In general, higher values for sperm quality parameters occur in the summer, in horses over four years old that are used sparingly [15]. In the horse breeding industry, the use of frozen semen has become increasingly important, despite its lower pregnancy rates compared with fresh or chilled semen. In fact, because breeding stallions are not usually selected according to their fertility or sperm ability for cryopreservation, stallions show a particularly high degree of individual variation with respect to the cryosurvival rate of their sperm [16,17]. Freezing–thawing processes are known to impair stallion sperm morphology, motility, and viability, as well as mitochondria integrity, thus altering the normal fertilizing ability. This is mainly due to osmotic stress and exposure to deep temperatures [18,19,20,21,22,23]. In equine sperm, different studies on the effects of centrifugation were performed, reporting contrasting results [24,25,26,27,28,29,30]. Although these studies investigated the effects of centrifugation on sperm motility, the effects on mitochondrial functionality and on different oxidative parameters have not been reported.

The role of mitochondria in sperm motility and quality has been emerging in recent years [31,32,33,34]. To be functionally active, mitochondria require an intact and impermeable inner membrane; in fact, MMP generated by proton pumps (Complexes I, III, and IV) is a key component in the ATP synthesis during oxidative phosphorylation [35]. Consequently, depolarization indicates mitochondrial damage and the inability of the cell to meet energy demands, whereas high hyperpolarization may lead to ROS production and cellular damage. Unlike other species, stallion sperm cells rely mostly on oxidative phosphorylation for their ATP production to be motile [36,37,38]. Consequently, in this species, depolarization of mitochondria is of particular concern as it may lead to reduction of sperm motility. The mitochondrion is the major ROS-producing organelle in the cell, with 1%–2% of the oxygen reduced during oxidative phosphorylation [37]. ROS production is necessary for normal sperm function, but it is also damaging in excess, causing oxidative stress. Consequences of oxidative stress are damaging effects on sperm structure and function including defects in mitochondrial respiration [39], lipid, protein and DNA damage, loss of motility, and apoptosis [37]. Due to the limited volume and restricted distribution of cytoplasmic space in which antioxidant enzymes are located, spermatozoa are highly vulnerable to oxidative attack. In particular, sperm membrane lipids are susceptible to oxidative stress since they are characterized by significant amounts of polyunsaturated fatty acids [40]. Their peroxidation results in formation of secondary products that cause oxidation of sperm proteins and consequent carbonylation, though a direct protein oxidation is also carried out by ROS [41]. Moreover, sperm chromatin integrity is essential as it represents half of the future embryo genome, and, after fertilization, oocytes may not be able to repair sperm DNA damage, especially in aged women [40,42]. Stallion sperm have been recently reported to produce high levels of ROS due to their intense mitochondrial activity and to have sophisticate systems to control redox homeostasis [38].

In this context, considering that information reported to date on the effects of centrifugation on sperm quality are controversial and mostly focused just on motility assessment, the aim of this study was to investigate the effects of different centrifugation protocols on cryopreserved stallion sperm motility and oxidative status in terms of oxygen consumption, MMP and oxidation-related injuries.

## 2. Materials and Methods

### 2.1. Chemicals

All chemicals were purchased from Sigma-Aldrich unless otherwise indicated.

### 2.2. Sperm Samples and Experimental Design

Equine sperm samples (0.4 mL/straw) from a single stallion of proven fertility and frozen at the concentration of 8 × 10^7^ sperm cells/mL, came from the Istituto Nazionale Fecondazione Artificiale, Cadriano, Granarolo Emilia, Bologna (Italy). Straws were rapidly thawed in water bath at 37 °C for 30 s and diluted in different solutions according to the experimental design. Subsequently, the total volume was split in 5 different tubes which were subjected to the following experimental conditions: i) no centrifugation (control); ii) centrifugation at 300× *g* for 5 min; iii) centrifugation at 300× *g* for 10 min; iv) centrifugation at 1500× *g* for 5 min; v) centrifugation at 1500× *g* for 10 min. Each experiment was performed in three independent replicates.

### 2.3. Sperm Motility Assessment by Computer-Assisted Sperm Analysis (CASA)

Sperm motility was analyzed by the HTM-IVOS Computer-Assisted Sperm Analyzer (CASA), (Hamilton-Thorne Biosciences, software version 12.3, Beverly, MA, USA). Sperm samples were resuspended (final concentration 30 × 10^6^ sperm cells/mL) in prewarmed (37 °C) sperm-Chatot, Ziomet and Bavister medium (sperm CZB medium; [43]). Then, 4 μL of sperm samples were loaded on specific slide chambers, 20 μm in depth (Leja 4, Leja Products B.V.) and analyzed at 37 °C. For each sample, 4 independent chambers were used and sperm samples included in 32 randomly chosen fields (8 fields/chamber) were counted. Analyses were performed with a 10× magnification objective and videos were recorded with 60 Hz frame rate and 45 frames/second. Mean numbers of cells analyzed for each condition were 915.917, 877.333, 985.667, 924.750, 1036.667 for control, 300× *g* for 5 min, 300× *g* for 10 min, 1500× *g* for 5 min, and 1500× *g* for 10 min respectively. The following parameters were measured: The percentage of motile spermatozoa (total motility, %); the percentage of progressively motile spermatozoa (progressive motility, %); the average path velocity (VAP, μm/s), calculated as the curvilinear trajectory of the sperm head; the straight-line rectilinear velocity (VSL, μm/s), calculated as the velocity of the sperm head along the trajectory between the first and the last spotted position; the curvilinear velocity (VCL, μm/s), calculated as the velocity of the sperm head along the total covered distance; the beat-cross frequency (BCF, Hz), the average rate at which the curvilinear path crosses the average path; two velocity ratios, straightness (STR) related to the linearity of the average path and linearity (LIN) related to the linearity of a curvilinear path, calculated as the ratio between VSL and VAP and between VSL and VCL, respectively; the amplitude of the lateral head displacement (ALH, μm). Furthermore, sperm velocity distribution was analyzed by identifying four sperm cell movement subcategories based on equine-specific VAP cut-off, as reported in the HTM-IVOS CASA system, version 12.3 software: percentage of rapid cells (fraction of cells moving with VAP > 50 μm/s; rapid, %); percentage of medium speed cells (fraction of cells moving with VAP values ranging from 20 to 50 μm/s; medium, %); percentage of slow cells (fraction of cells with VAP < 20 μm/s; slow, %) and percentage of static cells (fraction of cells not moving at all; static, %). Sperm motility parameters were defined as previously reported in the IVOS software manual and in [28,44].

### 2.4. Mitochondria Respiration Study

Sperm samples were subjected to hypotonic treatment for 1.5 h on ice as previously described [45]. Later on, samples were centrifuged at 800× *g* for 10 min and resuspended in isotonic salt medium (2 g/L bovine serum albumin, 113 mM KCl, 12.5 mM KH_2_PO_4_, 2.5 mM K_2_HPO4, 3 mM MgCl_2_, 0.4 mM ethylenediaminetetraacetic acid, and 20 mM tris adjusted to pH 7.4 with HCl). Oxygen uptake by hypotonically treated spermatozoa (10 × 10^6^ sperm cells/sample) was measured at 37 °C by using a Clark-type oxygen probe (Oxygraph, Hansatech Instruments) in the presence of mitochondrial respiratory substrates (10 mM pyruvate and 10 mM malate) and 0.76 µM of adenosine diphosphate (ADP). The rate of oxygen uptake by sperm mitochondria (V) was expressed as nmol O_2_ mL^−1^ × minute^−1^. The respiratory control ratio (RCR), an index of mitochondrial respiration efficiency, was calculated by dividing V3 (rate of oxygen uptake measured in the presence of respiratory substrates + ADP) by V4 (rate of oxygen uptake measured with respiratory substrates alone).

### 2.5. Sperm Staining with MitoTracker Green FM and Laser Scanning Confocal Microscopy (LSCM)-based Assessment of Mitochondrial Activity

Staining procedure with MitoTracker Green FM (M7514, Thermo Fisher Scientific) and Laser Scanning Confocal Microscopy (LSCM) assessment of mitochondrial activity were performed as previously described by Moscatelli et al. [33]. Sperm samples (5 × 10^6^ sperm cells/sample) were resuspended in prewarmed (37 °C) PBS containing 200 nM of the probe and incubated for 15 min at 37 °C. The cell-permeant MitoTracker probe contains a mildly thiol-reactive chloromethyl moiety for labelling mitochondria and it is distributed in sperm inner mitochondrial membrane proportionally to mitochondria activity [33,46]. Samples were analyzed by a confocal laser microscope (LEICA TCS SP8 X) and images were captured with the LasX Software using a 100× oil-immersion objective. For each sample, 6 fields were randomly chosen and the average projection of all images/field, was performed with the open source software ImageJ. In each single spermatozoon, fluorescence intensity related to mitochondrial membrane potential was quantified with the open source software ImageJ, after drawing a limited area around each midpiece.

### 2.6. Carbonylated Protein Assessment by Western Blot

Sperm sample were subjected to hypotonic sperm treatment (as described above) and three freezing/thawing cycles. The total amount of proteins was then quantified by the Bradford protein assay (Bio-Rad). The volume corresponding to 20 µg of proteins from sperm lysates was resuspended in sample buffer and used for oxidative protein damage evaluation by quantifying carbonyl groups on protein side chains using the OxyBlot TM Protein Oxidation Detection Kit (Merck Millipore), according to the manufacturer’s instructions and as previously reported by De Riccardis et al. [47]. Briefly, carbonyl groups were derivatized to 2,4-dinitrophenylhydrazone (DNP) by reaction with 2,4-dinitrophenylhydrazine (DNPH). Non-derivatized samples were used as negative controls. Both derivatized and nonderivatized samples were separated by 12% sodium dodecylsulphate–polyacrylamide gel electrophoresis (SDS-PAGE) and transferred by the Trans-Blot Turbo Transfer System (Bio-Rad) onto a nitrocellulose membrane. Membranes were blocked with 1% BSA in PBS, 0.1% Tween 20, incubated with a primary antibody solution against DNP-modified carbonyl groups (code: 90451; 1:150 dilution) and then with goat anti-rabbit/horseradish peroxidase secondary antibodies (code: 90452; 1:300 dilution). Protein-antibody complexes were identified with Clarity TM Western ECL Substrate (Bio-Rad) and the densitometric analyses of blots were performed with the open source software ImageJ.

### 2.7. Terminal Deoxynucleotidyl Transferase-mediated dUTP Nick-End Labeling (TUNEL) Assay for DNA Fragmentation Assessment

Sperm DNA fragmentation was assessed by using the Click-iT Plus TUNEL Assay (C10617, Thermo Fisher Scientific), following the manufacturer’s instructions. Briefly, sperm sample (5 × 10^6^ sperm cells/sample) were fixed in 4% PBS-buffered paraformaldehyde for 15 min at room temperature. Fixed spermatozoa were centrifuged (300× *g* for 5 min) to remove the fixative and then permeabilized with 0.25% Triton X-100 for 20 min. Samples were centrifuged again and washed twice with deionized water. Subsequently, 25 µL of each sample were spotted on a poly-l-lysine-coated glass slide and air dried. Sperm samples were incubated at 37 °C in a humidified chamber with TUNEL reagents consisting of the terminal deoxynucleotidyl transferase (TdT) enzyme, EdUTP nucleotides and the Alexa Fluor 488 picolyl azide dye. After incubation, sperm samples were washed with 3% Bovine Serum Albumin (BSA) in PBS. Total sperm nuclei were stained with 5 μg/mL Hoechst 33258 in 3:1 (v/v) glycerol/PBS, mounted on microscope slides, covered with cover-up micro slides, sailed with nail polish and kept at 4 °C in the dark until observation. Positive and negative controls were included in each replicate. Spermatozoa were observed by using a 63 × oil-immersion objective under a confocal laser microscope (LEICA TCS SP8) equipped with a 405 nm continuous wave diode laser for sperm nuclei observation (blue fluorescence) and a 488 nm continuous wave Argon laser for TUNEL positive cells observation (green fluorescence). Fluorescent emission was detected in the spectral window between 420 nm and 480 nm and between 500 nm and 550 nm, for Hoechst 33258 and Alexa Fluor 488 respectively. For each sample, 5 randomly fields were analyzed. DNA fragmentation index (DFI) was determined as the percentage of labelled cells (TUNEL positive) to the total cell number (Hoechst 33258) as previously reported by Hoogewijs et al. [28].

### 2.8. Statistical Analysis

All data are reported as mean value ± standard deviation, except for DFI values reported as percentage. P-values were calculated through the one-way ANOVA followed by Tukey’s post hoc test (PRISM software version 7.0; GraphPad, San Diego, CA, USA) to compare MitoTracker Green FM fluorescence intensity signals, motility parameters, RCRs and densitometric values between each treatment group and control. Percentages of spermatozoa showing DNA fragmentation were compared among groups by Chi-square test. Differences were considered significant when *p* < 0.05.

## 3. Results

### 3.1. Effects of Different Centrifugation Protocols on Sperm

**Figure 1:**
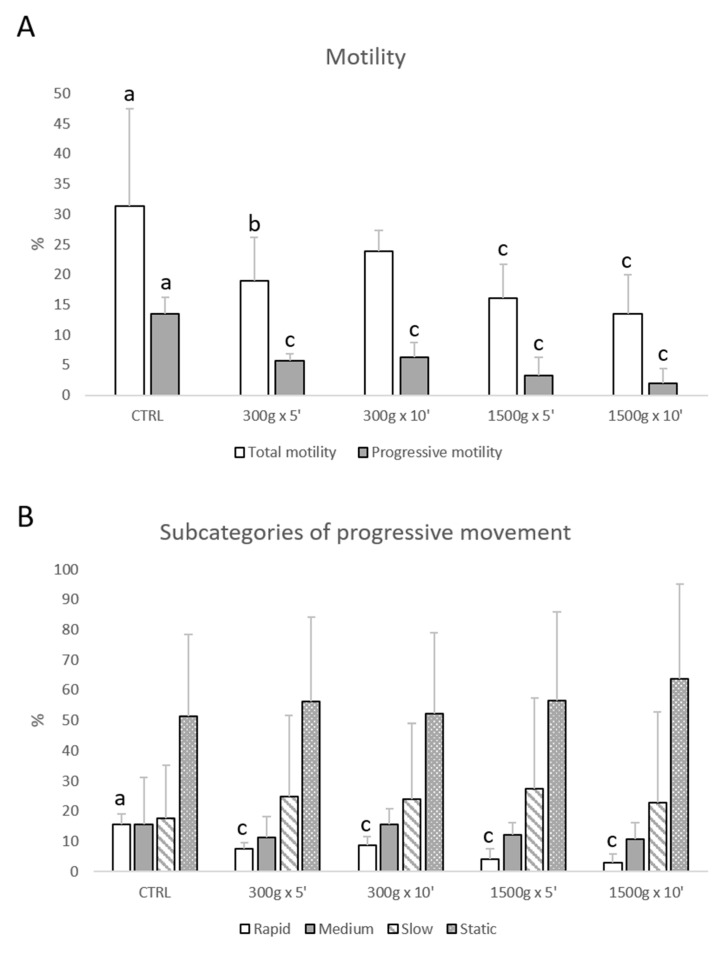
Effects of different centrifugation protocols on total and progressive motility of equine spermatozoa analyzed by Computer-Assisted Sperm Analysis (CASA). The reduction of the sperm motility was observed by analyzing total and progressive motility (panel A) and four subcategories of progressive movement: rapid, medium, slow, and static (panel B). One-way ANOVA followed by Tukey’s post hoc test: a,b = *p* < 0.05 a,c = *p* < 0.0001. Different superscripts indicate statistically significant differences.

Total motility was impaired at almost any tested centrifugation protocol (*p* < 0.05, for 300× *g* for 5 min vs. control; *p* < 0.0001, for 1500× *g* for 5 and 10 min respectively vs. control; Figure 1A), whereas progressive motility was impaired at any tested centrifugation protocol (*p* < 0.0001 vs. control; Figure 1A). Furthermore, by examining samples into four subcategories of progressive sperm movement (rapid, medium, slow, and static), it was possible to observe that only the rate of rapid cells was significantly reduced at any tested centrifugation protocol (*p* < 0.0001 vs. control; Figure 1B).

**Figure 2:**
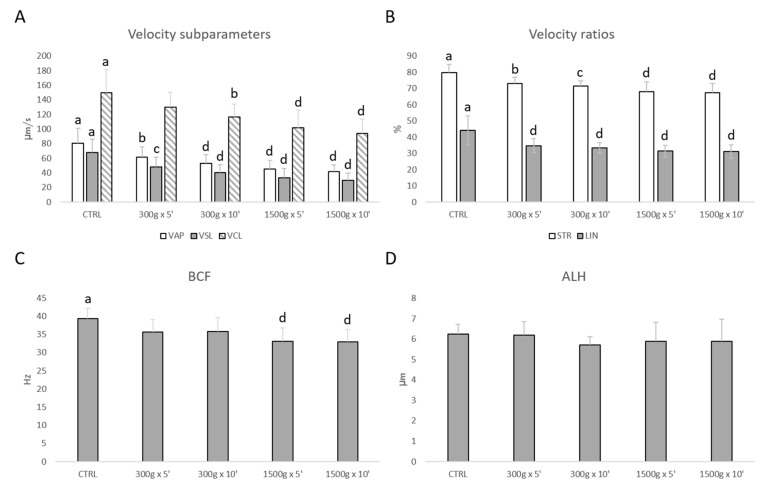
Effects of different centrifugation protocols on velocity subparameters (VAP, VCL, VSL; panel **A**), velocity ratios (STR and LIN; panel **B**), BCF (panel **C**) and ALH (panel **D**) in equine spermatozoa, as assessed by Computer Aided Sperm Analyzer (CASA). One-way ANOVA followed by Tukey’s post hoc test: a,b = *p* < 0.01; a,c = *p* < 0.001; a,d = *p* < 0.0001. Different superscripts indicate statistically significant differences.

Similarly, sperm velocity subparameters (VAP, VSL, VCL) were significantly reduced at almost any tested centrifugation protocol (Figure 2A). In details, VAP was significantly reduced after centrifugation at 300× *g* for 5 min (*p* < 0.01), at 300× *g* for 10 min and 1500× *g* for 5 and 10 min (*p* < 0.0001) respectively vs. control; VSL was significantly reduced after centrifugation at 300× *g* for 5 min (*p* < 0.001), at 300× *g* for 10 min and 1500× *g* for 5 and 10 min (*p* < 0.0001) respectively vs. control; VCL was significantly reduced after centrifugation at 300× *g* for 10 min (*p* < 0.01) and at 1500× *g* for 5 and 10 min (*p* < 0.0001) respectively vs. control. Additionally, velocity ratios were significantly reduced at any tested centrifugation protocols (Figure 2B). In particular, STR was significantly reduced after centrifugation at 300× *g* for 5 min (*p* < 0.01), at 300× *g* for 10 min (*p* < 0.001) and at 1500× *g* for 5 and 10 min (*p* < 0.0001) respectively vs. control; LIN was significantly reduced at any tested centrifugation protocol (*p* < 0.0001 vs. control). However, BCF was significantly reduced at the strongest tested centrifugation protocols (*p* < 0.0001 vs. control; Figure 2C), while ALH was not altered (*p* > 0.05 vs. control; Figure 2D).

For any motility parameter analyzed, no significant differences were found between samples centrifuged at the same centrifugal force and different times (300× *g* for 5 min vs. 300× *g* for 10 min and 1500× *g* for 5 min and 1500× *g* for 10 min, *p* > 0.05).

### 3.2. Effects of Different Centrifugation Protocols on Sperm Mitochondrial Respiration

**Figure 3:**
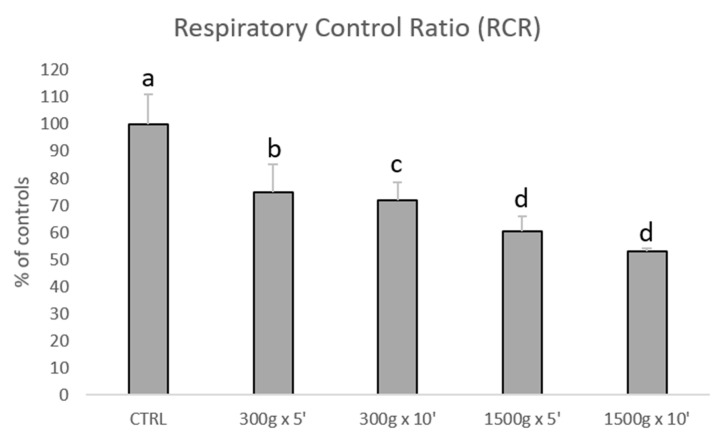
Bar graph showing the effects of different centrifugation protocols on the Respiratory Control Ratio (RCR) of equine spermatozoa. Data are shown as percentages of controls (RCR value for control samples was 1.9+0.2). One-way ANOVA followed by Tukey’s post hoc test: a,b = *p* < 0.05; a,c = *p* < 0.01; a,d = *p* < 0.0001.

Subsequently, we investigated the effects of different centrifugation protocols on RCR values, which are an index of mitochondrial respiration efficiency. RCR values were significantly reduced showing a linearity by increasing force and time of the centrifugation protocol (Figure 3). In details, we calculated the following RCRs: 1.9 ± 0.2 for control group, 1.4 ± 0.2 for 300× *g* for 5 min (*p* < 0.05 vs. control), 1.4 ± 0.1min for 300× *g* for 10 min (*p* < 0.01 vs. control) 1.2 ± 0.1 and 1.2 ± 0.0 for 1500× *g* for 5 and 10 min respectively (*p* < 0.001 vs. control). No statistical difference was found between samples centrifuged at the same centrifugal force and different times (300× *g* for 5 min vs. 300× *g* for 10 min and 1500× *g* for 5 min and 1500× *g* for 10 min, *p* > 0.05).

### 3.3. Effects of Different Centrifugation Protocols on Sperm Mitochondrial Membrane Potential

**Figure 4:**
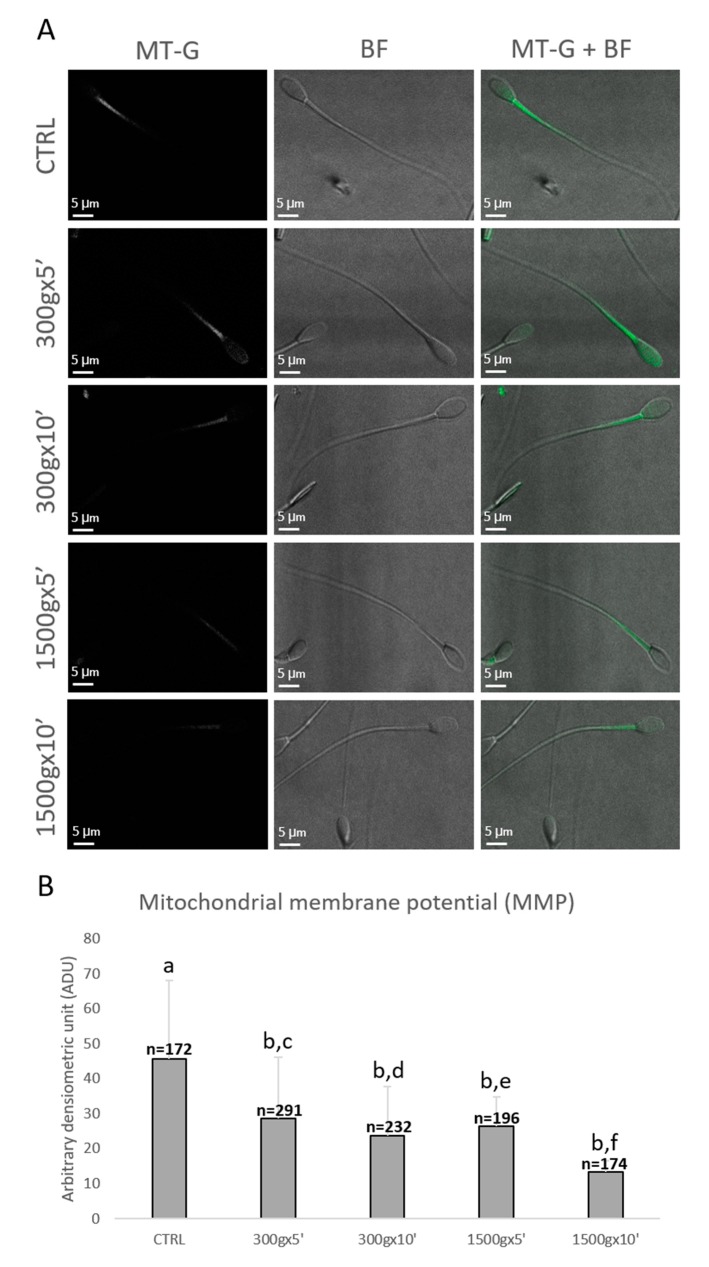
Representative photomicrographs of single equine sperm cells stained with MitoTracker Green FM (MT-G) after no centrifugation (CTRL) or centrifugation at 300× *g* for 5 min and 10 min and centrifugation at 1500× *g* for 5 min and 10 min. Corresponding confocal images showing MMP (column 1: MT-G), brightfield (column 2: BF) and merge (column 3: MT-G + BF) were reported. The average projection was obtained starting from a 0.5 μm-spaced z-stack acquired for an overall z-spanning of 2 μm. Scale bars were reported (panel **A**). Bar graph showing the effects of different centrifugation protocols on the Mitochondrial Membrane Potential (MMP) of equine spermatozoa. Data were shown as mean value ± standard deviation and expressed as Arbitrary Densitometric Units (ADU). Numbers of analyzed spermatozoa for each group are indicated at the top (panel **B**). One-way ANOVA followed by Tukey’s post hoc test: a,b = *p* < 0.0001; c,d = *p* < 0.0001; e,f = *p* < 0.0001.

Sperm mitochondrial functionality was further evaluated by LSCM after MitoTracker Green TM staining (Figure 4A). Specifically, numbers of spermatozoa analyzed for each group were *n* = 172, *n* = 291, *n* = 232, *n* = 196, *n* = 174 for control, 300× *g* for 5 min and 10 min, 1500× *g* for 5 min and 10 min, respectively. Similarly to sperm mitochondrial respiration, sperm MMP was significantly reduced at any tested centrifugation protocols in terms of fluorescence intensity (*p* < 0.0001 vs. control; Figure 4B). Interestingly, by comparing groups centrifuged at the same centrifugal force but different times, MMP significantly decreased after centrifugation at 300× *g* for 5 min vs. 10 min (*p* < 0.0001) and after centrifugation at 1500× *g* for 5 min vs. 10 min (*p* < 0.0001; Figure 4B).

### 3.4. Effect of Different Centrifugation Protocols on Sperm Oxidative Damages

**Figure 5:**
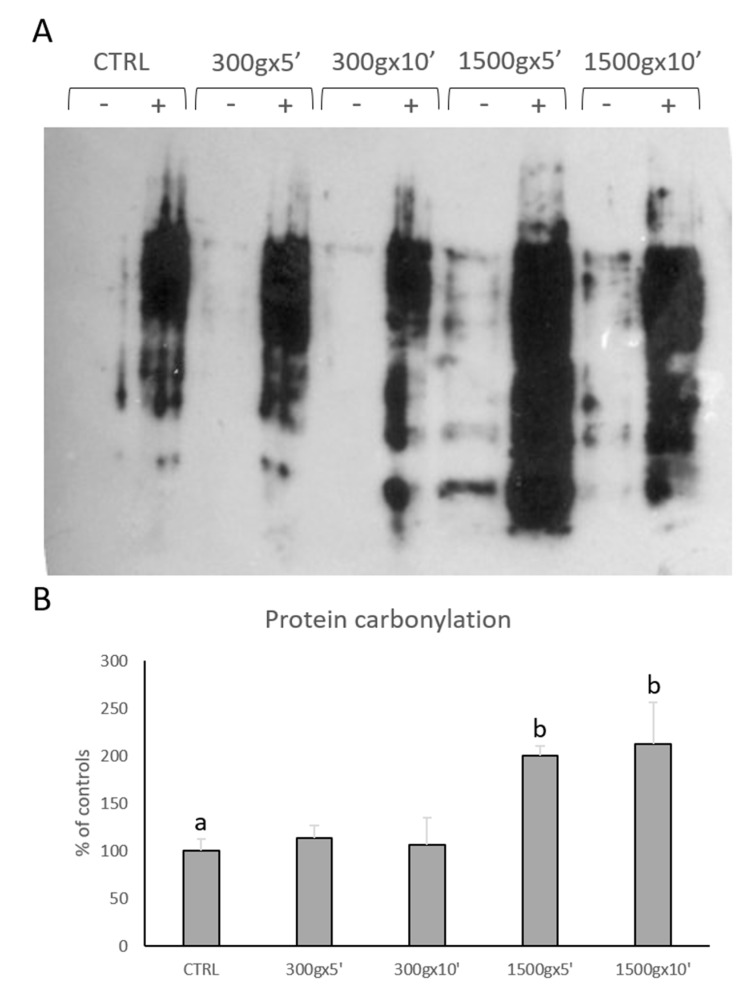
Representative OxyBlot without (-) and with (+) 2,4-dinitrophenylhydrazine (DNPH) derivatization of proteins extracted from equine sperm samples after no centrifugation (CTRL) or centrifugation at 300× *g* for 5 min and 10 min and centrifugation at 1500× *g* for 5min and 10 min (panel **A**). Bar graph showing the quantification analysis of protein carbonylation. Data are shown as percentages of controls (panel **B**). One-way ANOVA followed by Tukey’s post hoc test: a,b = *p* < 0.01. Different superscripts indicate statistically significant differences.

We performed OxyBlot analysis in order to determine post-translational modification of sperm cellular proteins due to oxidative stress (Figure 5A). Quantitative analysis of blots revealed significant increase in sperm protein carbonylation only at the strongest tested centrifugation protocols (*p* < 0.01; Figure 5B). No statistical difference was found between samples centrifuged at the same centrifugal force but different times (*p* > 0.05).

**Figure 6:**
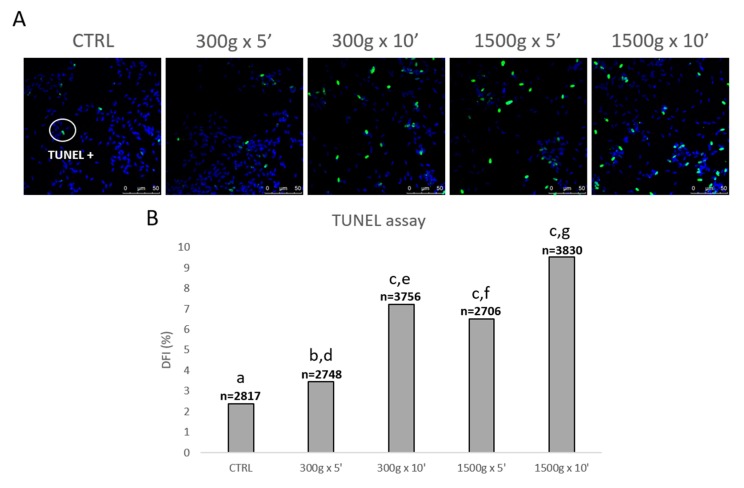
Representative photomicrographs of equine spermatozoa observed by Laser Scanning Confocal Microscopy (LSCM) after no centrifugation (CTRL) or centrifugation at 300× *g* for 5 min and 10 min and centrifugation at 1500× *g* for 5min and 10 min and Terminal Deoxynucleotidyl Transferase-mediated dUTP Nick-End Labeling (TUNEL) assay. Green and blue fluorescence were related to Alexa Fluor 488 and Hoechst 33258, respectively. Scale bars are reported (panel **A**). DNA fragmentation index (DFI) was determined as the percentage of green labelled cells (TUNEL positive) to the total cell number (Hoechst 33258). Numbers of analyzed spermatozoa for each group are indicated at the top (panel **B**). Chi-square test: a,b = *p* < 0.05; a,c = *p* < 0.0001; d,e = *p* < 0.0001; f,g = *p* < 0.0001.

In order to evaluate the effects of different centrifugation protocols on sperm DNA fragmentation, we performed the TUNEL assay (Figure 6A). At any tested centrifugation protocol, sperm DNA integrity was significantly impaired, with a less impact at the weakest centrifugation protocol tested (Figure 6B). In details, DFI significantly increased after centrifugation at 300× *g* for 5 min (*p* < 0.05), at 300× *g* for 10 min and 1500× *g* for 5 and 10 min (*p* < 0.0001) respectively vs. control. Interestingly, by comparing groups centrifuged at the same centrifugal force but different times, DFI significantly increased after centrifugation at 300× *g* for 5 min vs. 10 min (*p* < 0.0001) and after centrifugation at 1500× *g* for 5 min vs. 10 min (*p* < 0.0001; Figure 6B).

## 4. Discussion

Conventional sperm selection techniques, such as SU and DGC, require centrifugation steps for sperm processing, for subsequent andrological analysis or ARTs procedures. Studies in different species were carried out, particularly in humans, to investigate the effects of such techniques on sperm cells quality [48], though few studies have been performed to date to clarify whether centrifugation “per se” could impair sperm quality. Studies reporting the effects of centrifugation on equine spermatozoa have been carried out for decades and findings are controversial [24,25,26,27,28,29,30].

The present study analyzed the effects of different centrifugal forces and times on equine sperm quality, in terms of motility, examined by means of all parameters observable by CASA, and oxidative status, as the effects on mitochondrial functionality and different oxidative parameters have not been previously examined. In detail, we investigated the effects of centrifugation protocols with a weak centrifugal force (300 ×g), widely used to process sperm samples for ICSI [43,49], and two different times (5 and 10 min) and the effects of a very strong centrifugal force (1500 ×g) with the same times, considered as positive control, as generally, by increasing centrifugation time and force, a higher number of spermatozoa are pelleted.

As a first analysis, we evaluated sperm motility by CASA, and we found that almost any motility parameter was altered after centrifugation, including progressive motility and percentage of rapid cells useful for sperm fertilizing ability and VCL, VAP, LIN and STR that give information about sperm linearity and hyperactivation related to capacitation. Different results were obtained in previous studies in the stallion. Hoogewijs et al. found that, compared to uncentrifuged samples, centrifugation of semen resulted in a better sperm quality [28]. Len et al. found that neither total nor progressive motility were impaired when extended equine semen was centrifuged at 400× *g* or 900× *g*, while these parameters were altered after centrifugation at 4500× *g* [29]. These controversial results could be at least in part explained by the use of cryopreserved sperm in our study whereas chilled sperm was used in the studies by Hoogewis et al. and Len et al. Likely, this could be also the reason why we observed a reduction in motility parameters even at the weakest centrifugation protocol tested. In a study carried out on rat sperm, progressive motility was significantly reduced at different tested centrifugation protocols [10]. Another study carried out in the rat showed a reduction in sperm total motility after centrifugation at 600× *g* for 10 min and in progressive motility after centrifugation at 400× *g* and 600× *g* for 10 min [11]. These results may suggest that there are species-specific differences in sperm cell sensitivity or resistance to centrifugation as well as to cryopreservation, possibly due to the plasma membrane composition [50]. Furthermore, differences in sperm cryotolerance can be related also to interindividual variability. In particular, in stallion spermatozoa, it has been reported that membrane cholesterol content differences between stallions can affect cryosurvival [21], as well as the specific activity of superoxide dismutase in the seminal plasma of a given stallion ejaculate [22] and the ability of sperm from a specific ejaculate to resist and remain viable upon ROS levels increase [23].

Sperm mitochondria have been recently indicated as biomarkers of sperm health and fertility [37], though their status after centrifugation has not been studied yet in the stallion. For this reason, we investigated mitochondria functionality after centrifugation in two different ways: i) In a whole sperm population to obtain information about mitochondria bioenergetics; ii) in each single spermatozoon to obtain information about MMP. To the best of our knowledge, this is the first study on stallion sperm evaluating the effect of centrifugation on mitochondrial oxygen consumption in a whole sperm population by means of a polarographic assay. We found a progressive decrease in sperm mitochondria respiration from the gentlest centrifugation protocol used to the strongest. A positive correlation between mitochondrial oxygen consumption and sperm motility was previously shown in human samples by Ferramosca et al. [39]. In stallion sperm, mitochondrial oxygen consumption measurement has been already used in the context of cryopreservation [51] and aging [52]. These authors also found that mitochondrial oxygen consumption was positively correlated with equine sperm motility and viability parameters, suggesting that this kind of assay may be useful as a sensitive indicator of sperm health also in this species. Interestingly, a correlation between mitochondrial oxygen consumption and sperm motility was also found in previous studies in bull [53] and human sperm [54], suggesting that sperm, subjected to specific centrifugation conditions might shift their metabolic pathways to glycolysis. These studies, in agreement with our data, demonstrated by magnetic resonance spectroscopy or a metabolic assay platform, that the best sperm population in terms of motility is also the best in terms of metabolic rate. Subsequently, we analyzed mitochondria activity by LSCM in each single equine sperm cell after MitoTracker Green FM staining even for the first time, as in a previous publication in human spermatozoa it was shown that MitoTracker Green FM fluorescence was correlated to MMP [33], while other studies carried out in the stallion, used different probes and performed flow cytometry [55,56,57]. Thus, to the best of our knowledge, this is the first study investigating the effect of centrifugation “per se” on MMP of equine spermatozoa. Interestingly, we found a significant reduction in MMP at any tested centrifugation protocol, but also a time-dependent effect on this reduction with the same centrifugal force used. These data are quite in line with the results reported by Kim et al. who found a reduction in MMP after centrifugation at 400× *g* and 600× *g* for 10 min in rats sperm cells and after JC-1 staining [11]. As described above (see Introduction), it has been shown that equine spermatozoa rely almost entirely on oxidative phosphorylation for ATP production used for motility [36,37,38]. This suggests that if mitochondria are depolarized, the cell is unable to meet energy demands causing reduction of motility. Our results are in agreement with these observations, as reduced MPP was found to be related to reduced motility.

Aberrant mitochondria function may lead to enhanced ROS formation which in turn may damage cell molecules such as proteins and DNA [58]. For this reason, we analyzed two different parameters related to oxidative stress: Protein carbonylation and chromatin fragmentation. Protein carbonylation may be a direct index of oxidative stress, as it may lead to oxidation of proteins involved in sperm capacitation, motility and fertilizing ability [41]. To the best of our knowledge, only one study analyzed protein carbonylation in equine sperm [59]. These authors aimed to understand the correlation between protein carbonylation and stallion fertility. Thus, the our one is the first study analyzing protein carbonylation as an effect of centrifugation-induced sperm damage. In our experiments, increased protein carbonylation was found only at the highest centrifugal force tested. Kim et al. instead, found increased basal intracellular ROS in the total rodent sperm population after 400xg and 600xg for 10 min, suggesting a possible oxidative damage [11]. However, these authors directly measured ROS production, while we examined a delayed consequence of oxidative stress. DNA damage as single or double-strand breaks is a direct consequence of oxidative damage caused by hydrogen peroxide on sperm DNA, or may arise from lipid peroxidation by-products. In particular, the oxidative base adduct, 8-hydroxy-2′-deoxyguanosine (8OHdG) is known to be clearly identified by TUNEL [60]. Sperm cells only have one glycosylase in the base excision repair pathway, the 8-oxoguanine DNA glycosylase (OGG1), whose role is the active excision of 8OHdG, releasing this base adduct into the extracellular space. However, spermatozoa do not possess the downstream components of this DNA repair, thus causing base loss that destabilizes the ribose-phosphate backbone, leading to a strand break [40]. Spermatozoa with damaged chromatin are still motile and able to fertilize but may impair subsequent embryo development [40]. Unlike protein carbonylation, we found increased DFI at any tested centrifugation protocol, but also a time-dependent effect on this increase with the same centrifugal force used, though these findings are not in line with those of Hoogewijs et al., who showed that centrifugation did not influence DNA integrity [28]. Even in this case, these opposite results could be due to the use, in our experiments, of a cryopreserved sperm sample. Furthermore, sperm membrane is rich in polyunsaturated fatty acids susceptible to lipid peroxidation damages and accumulation of reactive by-products on sperm surface, that are released into cellular environment by the activity of phospholipase A2 to alter other proteins and DNA [41]. This biological mechanism may explain why we did not find an increase in protein carbonylation after sperm centrifugation at 300× *g* but we observed an increase in DFI.

## 5. Conclusions

Taken together, our data suggest that centrifugation, even weak and brief, impairs different aspects of stallion sperm functionality with negative impact on its fertilizing ability. Moreover, damage due to centrifugation is particularly severe in sperm samples with initial low quality. Even if a possible solution could be represented by the use of lower g forces, previous studies in other species demonstrated that centrifugation forces lower than those used in the present study, even not affecting sperm motility and MMP [11], induce high loss of cells in the supernatant [61] where substantial sperm cell loss is a big problem in ARTs. These results are noteworthy in equine reproduction and may have translational relevance in other animal species and in human ARTs. Non centrifugation based techniques should be proposed in order to identify new tools in the selection of good-quality spermatozoa in ARTs, in terms of motility but also mitochondrial activity and oxidative status. Indeed, microscopy based-, interaction based-, magnetic activated sperm sorting and, in particular, recently developing microfluidic devices are considered as cutting-edge and promising sperm selection techniques.
